# Mind the gap between low- and middle-income countries (LMICs) and high-income countries (HICs): fostering pediatric anesthesia globally

**DOI:** 10.1016/j.bjane.2024.844544

**Published:** 2024-07-30

**Authors:** Vinícius Caldeira Quintão, Ricardo Vieira Carlos, Gabriel Soares de Sousa, Maria José Carvalho Carmona

**Affiliations:** a Universidade de São Paulo, Faculdade de Medicina, Hospital das Clínicas (HCFMUSP), Instituto da Criança e do Adolescente, São Paulo, Brazil; b Universidade de São Paulo, Faculdade de Medicina (FMUSP), Disciplina de Anestesiologia, São Paulo, SP, Brazil

Pediatric anesthesia is a critical specialty within the broader field of anesthesiology, essential for ensuring the safety and effectiveness of surgical and procedural care for children. This domain is distinguished by its unique challenges and complexities, demanding specialized training and a profound understanding of pediatric physiology and pharmacology. Despite its importance, there are significant disparities in the training and practice of pediatric anesthesia, especially between low- and middle-income countries (LMICs) and high-income countries (HICs),^1^ and addressing them is crucial to improve global pediatric care and its outcomes.

The practice of pediatric anesthesia requires an intricate approach due to the physiological differences between children and adults. Children's anatomical and physiological characteristics, such as their airway size, cardiovascular function, and metabolic rate, need tailored anesthetic techniques and drug dosages.^2^ Additionally, pediatric patients often have distinct psychological needs, which can impact their response to anesthesia and their overall perioperative experience.^3^

Inadequate training in pediatric anesthesia can lead to significant challenges. Insufficient exposure and education during residency may contribute to the discomfort and uncertainty anesthesiologists might feel when managing pediatric cases. This lack of confidence can compromise patient safety and the effectiveness of anesthesia, particularly in high-complexity scenarios involving critically ill or high-risk pediatric patients.

The disparity between LMICs and HICs training in pediatric anesthesia is significant. In many LMICs, such as Brazil, pediatric anesthesia is not recognized as a formal subspecialty.^4^^,^^5^ Medical residency programs in these countries are often shorter when compared to most HICs, with limited time dedicated to pediatric anesthesia. Módolo et al. highlight that in Brazil, for instance, the relatively short duration of residency programs results in limited training focused on pediatric anesthesia, often confined to the third year of residency.^4^ This shortfall in training can severely impact the quality of care provided to pediatric patients, posing significant risks to their safety and the efficacy of surgical procedures.

In contrast, many HICs have established longer and more specialized training programs that provide a comprehensive education in pediatric anesthesia.^6^ These programs are designed to cover all aspects of pediatric anesthesiology, ensuring that practitioners are well-prepared to manage children's unique needs. They not only enhance the proficiency of anesthesiologists but also contribute to better perioperative outcomes for pediatric patients. As a valuable educational tool, Braz et al. presented an update on the mechanisms and risk factors of anesthesia-related cardiac arrest in pediatric surgical patients in countries with different human development indexes and over time. This study promotes a better understanding of these events and differences between countries.^7^

Recent advancements in pediatric anesthesia have brought significant improvements in practice. Innovations in techniques and approaches have enhanced the safety and effectiveness of pediatric anesthetic care. For instance, Abdel-Chaffar et al. analyzed the relationship between EtCO_2_ and PaCO_2_ over time in elective pediatric laparoscopic surgeries to maintain normocapnia in children. However, they found that capnography as a trend monitor in pediatric laparoscopic surgeries may not reliably indicate PaCO_2_ levels, highlighting the need for further research into noninvasive monitoring of PaCO_2_.^8^ In this special issue, Oliveira et al. demonstrated that the use of intracuff lidocaine associated with intravenous dexamethasone can be effective in reducing sore throat 24 hours after tonsillectomy and adenoidectomy when compared to the use of air to inflate the cuff.^9^

Another area of advancement is regional anesthesia. Peripheral nerve blocking in children is recently growing in evidence. Kamal et al. demonstrated the efficacy of real-time ultrasound-guided epidural catheter placement in infants.^10^ This technique allows for more precise placement of epidural catheters, which can improve pain management and procedural outcomes in young patients. Still, deciding which block is most effective in specific surgical scenarios can be challenging. Mutlu et al. compared the posterior transversus abdominis plane block versus lateral quadratus lumborum block in children undergoing open orchiopexy, offering more insight into pain management in this frequently performed surgery.^11^ Yayik et al. showed that the costoclavicular approach to the brachial plexus is as effective as the lateral sagittal approach, with the advantage of shorter time to perform the block.^12^ Similarly, Vinent et al. explored the use of ultrasound-guided nerve blocks in pediatric patients with complex regional pain syndrome, showcasing how technological advancements can improve pain management and overall patient outcomes.^13^

Another important progress is the comparison of premedication strategies. Pereira et al. conducted a comprehensive meta-analysis comparing intranasal dexmedetomidine and oral midazolam for premedication in pediatric patients.^14^ Their findings provide valuable insights into which premedication method may offer better efficacy and safety, thereby helping refine preoperative practices and reduce anxiety in pediatric patients. Another innovative approach to reducing anxiety examined the feasibility of the virtual presence of parents during anesthetic induction. This method has been shown to significantly reduce preoperative anxiety, offering a new possibility to add comfort for pediatric patients.^15^ Following the same reasoning, Carneiro et al. showed that information prior to surgery regarding the anesthetic act may be well accepted by children and caregivers when presented as a comic strip leaflet.^16^ Still looking for a better patient experience, Ng et al. presented a meta-analysis in which the administration of nalbuphine is associated with a significant decrease in the incidence of emergence delirium and postoperative pain scores among children undergoing surgery.^17^

The role of advanced techniques in pediatric anesthesia is further highlighted by Sahutoglu et al., who reported on the management of transfusion-related acute lung injury using high-flow oxygen therapy in a pediatric patient.^18^ This case underscores the importance of innovative solutions in addressing complex clinical scenarios that can arise during pediatric anesthesia. Han et al. also contributed to the field by investigating preoperative gastric volume assessment using ultrasound in cerebral palsy pediatric patients, offering new insights into optimizing preoperative care.^19^ In the same way, Abdel-Ghaffar et al. evaluated the perfusion index derived from pulse oximetry as a tool for assessing anesthetic depth in comparison with the index derived from evoked potentials, demonstrating possible new use for an already known parameter.^20^

To bridge the gap in pediatric anesthesia training, it is essential to adopt a global perspective and address systemic challenges. Mumphansha et al. explored the influence of power and privilege in pediatric anesthesia, showing these factors can affect training opportunities and access to advanced care.^21^ Their analysis highlights the need for more equitable distribution of resources and training opportunities to make sure all practitioners, regardless of their location, have access to high-quality education and support.

Matava and Kurth provide valuable insights into global perspectives on pediatric anesthesia, emphasizing the importance of international collaboration and knowledge exchange.^6^ Their editorial highlights the benefits of integrating best practices from HICs and adapting them to the local context in LMICs. By fostering international partnerships, LMICs can enhance their training programs, improve care quality, and ultimately achieve better perioperative outcomes for pediatric patients. Similarly, Johansen et al. carried out a retrospective study that analyzed the impact of gestational age on gastroesophageal and respiratory complications. In addition to suggesting that gastrointestinal complications might be related to gestational age, they also propose that multicenter collaborations are essential for this type of condition.^22^

The role of international collaborations is crucial in addressing the disparities in pediatric anesthesia training. Sharing expertise, resources, and experiences across borders can help develop standardized guidelines and protocols that improve the overall quality of pediatric anesthesia care. These collaborations, especially observational and pragmatic studies, can also facilitate the exchange of innovative techniques and approaches, further advancing in the field and enhancing patient safety.

To effectively bridge the gap in pediatric anesthesia training and practice, several strategies should be implemented. First, developing and implementing comprehensive, specialized training programs for pediatric anesthesia is essential. These programs should offer extensive hands-on experience and exposure to a variety of pediatric cases, ensuring that practitioners are well-prepared to manage complex scenarios.

Second, fostering international collaborations and partnerships, especially between HICs and LMICs, can play a pivotal role in improving pediatric anesthesia training. By sharing knowledge, resources, and best practices, anesthesiologists from different regions can learn from each other and adopt effective strategies to enhance their own training programs and clinical practices.

Third, investing in research and innovation is crucial for advancing pediatric anesthesia practices, given that there is relatively less innovation in pediatric anesthesia compared to adult medicine. Continued research can provide valuable insights into new techniques, technologies, and approaches that can improve patient outcomes and safety. Encouraging and supporting research initiatives in both HICs and LMICs can help drive progress and address the challenges faced in pediatric anesthesia. As an example, Özel et al. study on the Pediatric Early Warning Score (PEWS) highlights the potential for predictive tools to improve the prognosis of critical pediatric trauma patients, furthering this research agenda.^23^

This Pediatrics Special Issue of the *Brazilian Journal of Anesthesiology* serves as an important platform for addressing the current state of pediatric anesthesia training and practice, particularly in LMICs. This issue brings together a diverse collection of articles that highlight advances, challenges, and needs within the field. By promoting comprehensive discussions and offering valuable insights, it aims to contribute to the improvement of pediatric anesthesia training and practice.

The articles featured in this edition provide a broad perspective on the state of pediatric anesthesia, ranging from innovative techniques and approaches to systemic challenges and global perspectives. These contributions are instrumental in advancing the field and addressing the disparities that exist between LMICs and HICs.

In conclusion, addressing the disparities in pediatric anesthesia training and practice is crucial for ensuring the safety and well-being of pediatric patients. Developing specialized training programs, fostering international collaborations, and investing in research and innovation are essential steps in enhancing the quality of pediatric anesthesia care. By bridging the gap between low- and middle-income countries and high-income countries, we can improve perioperative outcomes and guarantee that children worldwide receive the highest standard of care. The ongoing efforts to advance pediatric anesthesia, as highlighted in this special issue of the *Brazilian Journal of Anesthesiology*, are vital for achieving these goals. Through continued collaboration, research, and the implementation of best practices, the field of pediatric anesthesia can evolve to meet the needs of patients across the globe and improve the overall quality of care for children.

## Declaration of competing interest

Vinícius Caldeira Quintão and Maria José Carvalho Carmona serve as Associate Editors of the Brazilian Journal of Anesthesiology. The other authors declare no conflicts of interest.
